# Epitope-Based Immunoinformatics Approach on Nucleocapsid Protein of Severe Acute Respiratory Syndrome-Coronavirus-2

**DOI:** 10.3390/molecules25215088

**Published:** 2020-11-02

**Authors:** Ahmed Rakib, Saad Ahmed Sami, Md. Ashiqul Islam, Shahriar Ahmed, Farhana Binta Faiz, Bibi Humayra Khanam, Kay Kay Shain Marma, Maksuda Rahman, Mir Muhammad Nasir Uddin, Firzan Nainu, Talha Bin Emran, Jesus Simal-Gandara

**Affiliations:** 1 Department of Pharmacy, Faculty of Biological Sciences, University of Chittagong, Chittagong 4331, Bangladesh; rakib.pharmacy.cu@gmail.com (A.R.); s.a.sami18pharm@gmail.com (S.A.S.); ashiqulanik@gmail.com (M.A.I.); shahriarahmed65@gmail.com (S.A.); farhanafaiz037@gmail.com (F.B.F.); humayra.Khanam@cu.ac.bd (B.H.K.); kkshainapu@gmail.com (K.K.S.M.); maksudarahman777@gmail.com (M.R.); nasirmir@cu.ac.bd (M.M.N.U.); 2 Department of Pharmacy, Mawlana Bhashani Science & Technology University, Santosh, Tangail 1902, Bangladesh; 3 Faculty of Pharmacy, Hasanuddin University, Tamalanrea, Kota Makassar, Sulawesi Selatan 90245, Indonesia; firzannainu@unhas.ac.id; 4 Department of Pharmacy, BGC Trust University Bangladesh, Chittagong 4381, Bangladesh; 5 Nutrition and Bromatology Group, Department of Analytical and Food Chemistry, Faculty of Food Science and Technology, University of Vigo–Ourense Campus, E32004 Ourense, Spain

**Keywords:** COVID-19, SARS-CoV-2, vaccine, nucleocapsid protein, bioinformatics, immunoinformatics, epitope

## Abstract

With an increasing fatality rate, severe acute respiratory syndrome-coronavirus-2 (SARS-CoV-2) has emerged as a promising threat to human health worldwide. Recently, the World Health Organization (WHO) has announced the infectious disease caused by SARS-CoV-2, which is known as coronavirus disease-2019 (COVID-2019), as a global pandemic. Additionally, the positive cases are still following an upward trend worldwide and as a corollary, there is a need for a potential vaccine to impede the progression of the disease. Lately, it has been documented that the nucleocapsid (N) protein of SARS-CoV-2 is responsible for viral replication and interferes with host immune responses. We comparatively analyzed the sequences of N protein of SARS-CoV-2 for the identification of core attributes and analyzed the ancestry through phylogenetic analysis. Subsequently, we predicted the most immunogenic epitope for the T-cell and B-cell. Importantly, our investigation mainly focused on major histocompatibility complex (MHC) class I potential peptides and NTASWFTAL interacted with most human leukocyte antigen (HLA) that are encoded by MHC class I molecules. Further, molecular docking analysis unveiled that NTASWFTAL possessed a greater affinity towards HLA and also available in a greater range of the population. Our study provides a consolidated base for vaccine design and we hope that this computational analysis will pave the way for designing novel vaccine candidates.

## 1. Introduction

The present world has witnessed the outbreak of many life-threatening human pathogens including Ebola, Chikungunya, Zika, severe acute respiratory syndrome coronavirus (SARS-CoV), and Middle East respiratory syndrome coronavirus (MERS-CoV) in the 21st century. More recently in late December 2019, a cluster of pneumonia cases was reported in the city of Wuhan, Hubei province, China, which was of unknown cause. Later it was confirmed that these pneumonia cases were due to a novel coronavirus named SARS-CoV-2 (previously named as 2019-nCoV) and the disease condition of this virus is referred to as COVID-19 [1,2,3]. On 11 March, 2020, the World Health Organization (WHO) assessed that COVID-19 can be characterized as a pandemic. The current COVID-19 pandemic is a global concern and is spreading at an alarming rate and as of 26 October, 2020, more than 43.2 million cases along with over 1.16 million deaths have been reported globally [4].

As COVID-19 is mainly a respiratory disease, in most cases it might affect the lungs only. The primary mode of infection is human-to-human transmission through close contact, which occurs via spraying droplets from the infected individual through their cough or sneeze. The symptoms of this coronavirus can be mild to moderate or severe including, fever, cough, and shortness of breath or pneumonia. Respiratory, hepatic and neurological complications can be seen in case of severe cases that can lead to death. It seems that the severity and fatality rate of COVID-19 is milder than that of SARS and MERS. Although diarrhea was presented in about 20–25% of patients with SARS and MERS, intestinal symptoms were rarely reported in patients with COVID-19 [5,6,7]. Multi-organ failure, especially in elderly people and people with underlying health conditions, such as hypertension, cardiovascular disease and diabetes, are exhibiting a higher mortality rate in COVID-19.

Interestingly, SARS-CoV-2 has 82% similarity with the original SARS-CoV virus attributed to the outbreak in 2003 [8]. A mature SARS-CoV-2 virus generally has a polyprotein (the open reading frame 1a and 1b, Orf1ab), four structural proteins such as envelope (E) protein; membrane (M) protein; nucleocapsid (N) protein; spike (S) protein and five accessory proteins (Orf3a, Orf6, Orf7a, Orf8 and Orf10), and, particularly, SARS-CoV-2 encodes an additional glycoprotein having acetyl esterase and hemagglutination (HE) attributes, which identified it distinct to its two predecessors [9]. The functions of accessory proteins may include signal inhibition, apoptosis induction and cell cycle arrest [10]. The S protein on the surface of the viral particle enables the infection of host cells by binding to the host cell receptor angiotensin-converting enzyme 2 (ACE2), utilizing the S-protein’s receptor-binding domain (RBD).

The N protein binds to the RNA genome of the COVID-19 and creates a shell or capsid around the enclosed nucleic acid. The N protein is involved in viral RNA synthesis and folding, which interacts with the viral membrane protein during viral assembly and affects host cell responses including cell cycle and translation. An epitope-based peptide vaccine has been raised in this aspect. The core mechanism of the peptide vaccine is based on the chemical method to synthesize the recognized B-cell and T-cell epitopes that can induce specific immune responses and are immune-dominant. T-cell epitopes are short peptide fragments (8–20 amino acids) while the B-cell epitopes can be proteins [11,12].

Once a mutated virus infects the host cells by escaping the antibodies, it then relies upon the T-cell mediated immunity to fight against the virus. Viral proteins are processed into short peptides inside the infected cells and then loaded onto major histocompatibility complexes (MHC) proteins. After that, the MHC-peptide complexes are presented on the infected cell surface for recognition by specific T-cells. Activated CD8^+^ T-cells then recognize the infected cells and clear them. T-cell immunity also depends strictly on the MHC-peptide complexes, which are similar to the antigen-antibody association. MHC proteins are encoded by human leukocyte antigen (HLA), which is located among the most genetically variable regions on the human genome. Each HLA allele can only present a certain set of peptides that can be presented on the infected cell surface and recognized by T-cells are called T-cell epitopes. For a vaccine, it is essential to identify T-cell epitopes that originate from conserved regions of the virus T cell responses against the S and N proteins have been reported to be the most dominant and long-lasting [13].

To develop effective diagnostic tests and vaccine, the identification of B-cell and T-cell epitopes for SARS-CoV-2 proteins are critical especially for structural N and S proteins. Both humoral immunity and cellular immunity provided by B-cell antibodies and T-cells respectively are essential for effective vaccines [14,15]. Although humans may mount an antibody response against viruses normally, only neutralizing antibodies can block the entry of viruses into human cells completely [16]. Antibody binding site’s location on a viral protein strongly affects the body’s ability to produce neutralizing antibodies [17]. It is important to understand whether SARS-CoV-2 has potential antibody binding sites (B-cell epitopes) near their interacting surface with its known human entry receptor, ACE2. Besides neutralizing antibodies, human bodies also depend on cytotoxic CD8^+^ T-cells and helper CD4^+^ T-cells to clear viruses completely from the body. For antiviral T-cell responses, presentation of viral peptides by human MHC class I and class II is essential [18]. MHC-I analysis includes common alleles for HLA-A, HLA-B and HLA-C. Multiple investigations have indicated that antibodies generated against the N protein of SARS-CoV are a highly immunogenic and abundantly expressed protein during infection [19].

**Figure 1:**
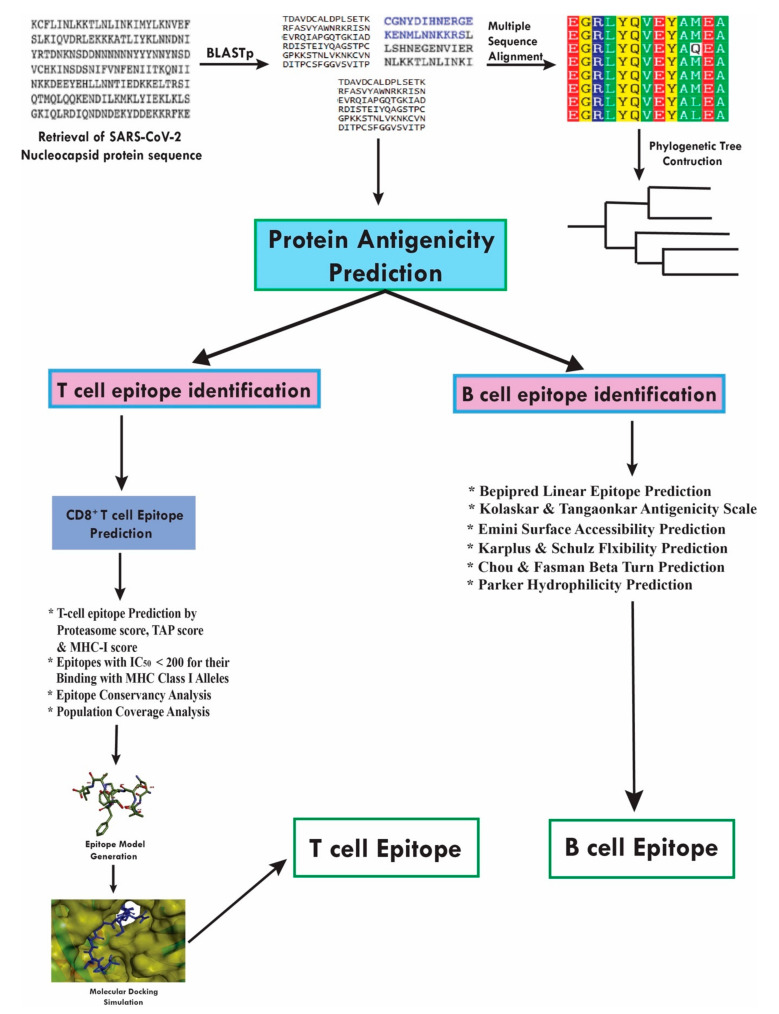
Workflow of the methodologies used in peptide vaccine design by utilizing SARS-CoV-2 nucleocapsid (N) protein.

Our group is targeting for immunoinformatics-based vaccine design using bioinformatics and immunoinformatics tools by utilizing different protein sequences of SARS-CoV-2. Recently, we have already established potential B and T-cell epitopes with a greater candidacy profile using the S protein of SARS-CoV-2 [20]. Moreover, other published work also utilized the S protein of SARS-CoV-2 for epitope-based vaccine design [21]. The purpose of our present study is to promote the designing of a vaccine against COVID-19 using in silico methods, considering SARS-CoV-2 N protein. The reason for focusing particularly on the epitopes in the N structural proteins is due to their dominant and long-lasting immune response, which was reported against SARS-CoV previously [22]. Besides, it has been reported that the N protein of many viruses are highly conserved and immunogenic, which expressed extensively in the course of infection [23]. Particularly, it has been reported recently that the N protein and E protein of SARS-CoV-2 are most evolutionarily conserved [24,25]. For the identified T-cell epitopes, we incorporated the information on the associated MHC alleles so that we can provide a list of epitopes that seek to maximize population coverage globally. Therefore, we designed an epitope-based peptide vaccine through utilizing the SARS-CoV-2 N protein (Figure 1) to potentially narrow down the search for potent targets against SARS-CoV-2 using the computational approach with an expectation that the wet laboratory research will validate our result.

## 2. Results

### 2.1. Sequence Retrieval and Analysis

We retrieved the SARS-CoV-2 N protein sequence from the NCBI database (Accession No.: QIC53221.1). Then we performed BLASTp using NCBI-BLAST for the N protein of SARS-CoV-2. We searched for a total of 100 homologs with >60% identical sequences. Multiple sequence alignment (MSA) was then performed to find out the conservancy among the target proteins. (Supplementary Data 1), and a phylogenetic tree was constructed to analyze the evolutionary divergence amongst them (Figure S1). From the results of the MSA analysis, it has been confirmed that the protein sequences have a close relationship.

### 2.2. Antigenic Protein Prediction

The most potent antigenic protein of SARS-CoV-2 N protein was predicted by VaxiJen v2.0, which is based on the auto-cross covariance transformation of protein sequences into uniform vectors of principal amino acid properties. The VaxiJen tool mainly encompasses the physicochemical properties of the protein sequence [26]. The overall antigen prediction score was 0.5002 (probable antigen) at a 0.4 threshold value.

### 2.3. Toxicity Prediction

Prediction of the toxicity of peptides before considering them, as epitopes are very important for saving both time and to make it cost effective. The toxicity of the selected peptide sequences was assessed using the ToxinPred web server. ToxinPred is a unique tool, which is based on support vector machine (SVM) in predicting toxicity of peptides and several physicochemical properties, including hydrophilicity, hydrophobicity, charge and molecular weight. The results from the ToxinPred tool showed that all of our probable epitopes were found non-toxic (Table 1).

### 2.4. Protein Structure Prediction and Validation

**Figure 2:**
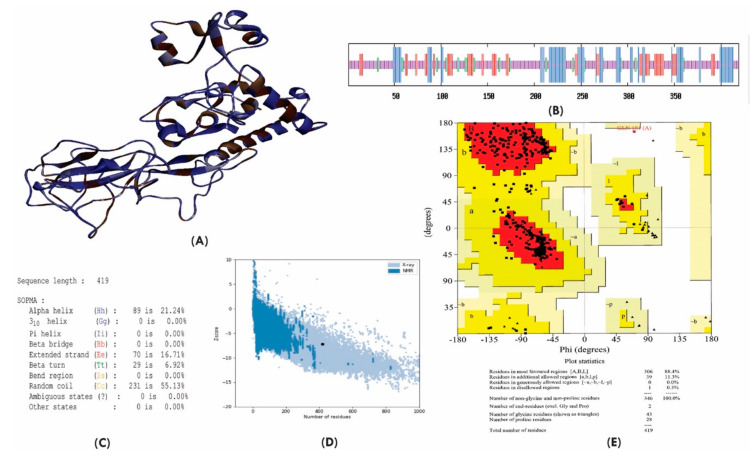
(**A**) 3D structure of modeled SARS-CoV-2 N protein; (**B**) composition of the secondary structure from amino acid residues of SARS-CoV-2 N protein; (**C**) predicted secondary structure of SARS-CoV-2 N protein; (**D**) Z-score of the SARS-CoV-2 N protein predicted by PROSA server and (**E**) Ramachandran plot analysis of the SARS-CoV-2 N protein.

The secondary structure of the SARS-CoV-2 N protein was predicted using the self-optimized prediction method with alignment (SOPMA), an online server, During prediction, the SOPMA server can be able to locate almost all of the stretches with the regular structure, which investigate the recognition of folding pattern in an efficient way [27]. The secondary structure of a protein describes mainly the α-helix, β-sheets and random coil. SARS-CoV-2 N protein has 419 residues (Figure 2A), of which 89 residues were remained in the α-helix, 70 residues were from the extended strand, 29 residues were observed in the β-sheets, and 219 residues were remained as random coil (Figure 2B,C). For 3D structure, we built a model using the Robetta online server. The Robetta server predicts the tertiary structure of a given protein from the inputted genomic data. The Robetta server utilizes a fully automated implementation of the Rosetta software package for the inference of the structural information of the protein [28]. In the current experiment, the Robetta server predicted five models for the SARS-CoV-2 N protein, which were validated using PROCHECK and PROSA-Z score. From the result of the validation, it has been observed that Model 4 predicted by the Robetta server have possessed 88.4% amino acid residues in the Rama favored region and delineated Z-score of −7.24, which depicted the model as a good quality model (Figure 2D,E). Although the Z-score for model 1 was shown −7.42, it possessed less amino acid residues in the Rama favored region (Figure S2). In addition, we analyzed the Ramachandran plot statistics and Z-score for the crystal structure of SARS-Cov-2 N protein (Resolution: 2.70 Å). The results showed that the Rama favored region for the crystal structure of SARS-CoV-2 N protein was 88.1% and Z-score was −5.06, which was less compared to the model structure (Figure S2). Hence, model 4 could be used for further analysis.

### 2.5. CD8^+^ T-Cell Epitope Identification

The NetCTL 1.2 server was utilized for the prediction of T-cell epitopes. The number of T-cell epitopes depended on the length of the sequence. Further the predicted epitopes with strong binding affinities were subjected to several immune filters in order to screen out the best possible epitopes, including conservation among the protein sequences included in the study, should be immunogenic, should be non-allergic and importantly should not overlap with any human proteins. Based on high combinatorial and MHC binding, the top eight epitopes were predicted by the NetCTL server from the selected protein sequence that was selected for further analysis. Using the MHC-I binding prediction tool, which is based on stabilized matrix method (SMM), we selected those MHC-I alleles for which the epitopes showed the highest affinity (half maximal inhibitory concentration, IC_50_ < 200 nm).

Proteasomes play an important role in cleaving the peptide bond, resulting in the conversion of protein into the peptide. The peptide molecules that are homogeneous to class I MHC molecules and the peptide-MHC molecule after the proteasomal cleavage were presented as T-helper cells after the transportation into the cell membrane. The total score of each epitope–HLA interaction was taken into consideration and higher processing efficiency was meant by obtaining a higher score. The epitope NTASWFTAL interacted with most of the MHC-I alleles including, HLA-A*68:02, HLA-C*16:01, HLA-C*03:03, HLA-C*03:04, HLA-C*12:03, HLA-A*02:06, HLA-C*03:02, HLA-A*26:01 and HLA-C*14:02 (Table 2). Moreover, the MHC-NP prediction tool was used to find the highest probable score of our predicted epitope NTASWFTAL, with a score of 1.11, for HLA-A*68:02. Furthermore, all the predicted epitopes had a maximum identity for conservancy hit and 100% maximum identity was found (Table 2). Additionally, the I-pMHC immunogenicity prediction analysis of the epitope NTASWFTAL was found 0.22775 (Table 2).

### 2.6. Population Coverage

Population coverage analysis is crucial in determining a peptide sequence as vaccine candidates. Accordingly, epitope-based vaccines can be designed to maximize the population coverage and minimizing the complexity regarding the variability of the population coverage observed in different ethnic groups. In the current study, the cumulative amount of the population coverage was obtained for the predicted epitope NTASWFTAL. Results from the population coverage demonstrated that with 57.16% coverage, East Asia found the highest coverage region. The results of the population coverage were shown in Table 3 and Figures S3–S6.

### 2.7. Allergenicity Assessment

The AllerTop server was used for the identification of the allergic reaction caused by a vaccine in an individual that might be harmful or life-threatening. The AllerTop server predicts allergenicity based on several factors, including, amino acid descriptors, accounting for residue hydrophobicity, size, abundance, helix- and β-strand forming propensities and a machine learning approach, namely the *k* nearest neighbors (*k*NN) method was implemented to classify allergens and non-allergens [29]. The allergenicity of the selected epitope was calculated using the AllerTop tool and predicted as a probable non-allergen.

### 2.8. Molecular Docking Analysis for HLA and Epitope Interaction

**Figure 3:**
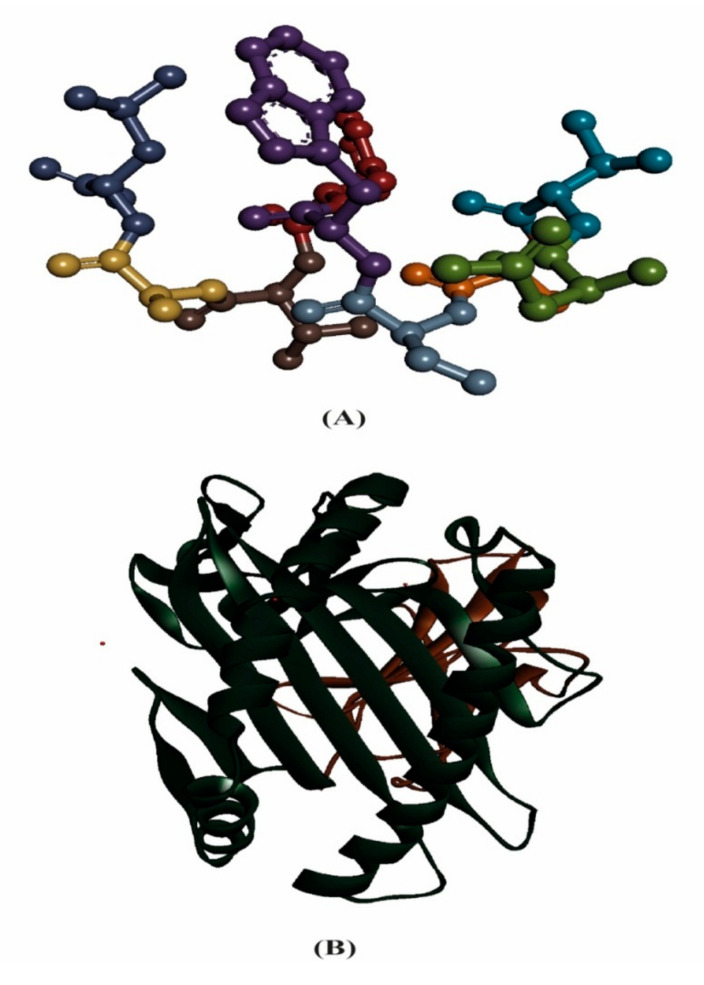
(**A**) Three-dimensional representation of the predicted epitope, NTASWFTAL, and (**B**) three-dimensional representation of the HLA-A*68:02 molecule.

Molecular docking analysis is used for the prediction of a ligand–receptor interaction. The advancement in computational biology techniques in the last few decades have allowed for further development in molecular docking algorithms for determining the flexibility of a protein and currently, molecular docking is considered as widespread tools used in computational biology techniques. In this study, the verification of the interaction between the HLA molecules and our predicted potential epitope was done by molecular docking simulation using AutoDock Vina in PyRx 0.8 software. Among all the MHC class I alleles, only HLA-A*68:02 had a maximum probable score for our most potent epitope NTASWFTAL. Therefore, we carried out the molecular docking study using HLA-A*68:02 (PDB ID: 4I48). The 3D structure of the predicted epitope, NTASWFTAL and HLA-A*68:02 molecules are represented in Figure 3.

**Figure 4:**
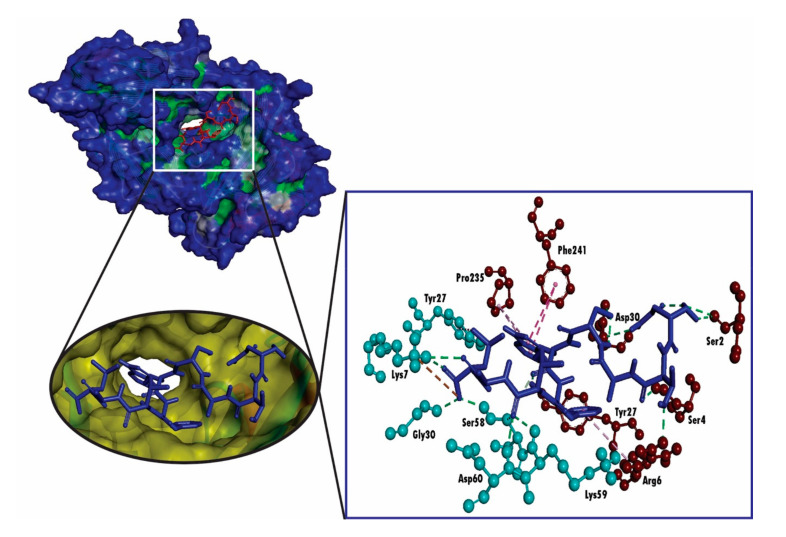
3D representation of molecular docking studies representing the binding affinity of the predicted epitope, NTASWFTAL to the groove of the HLA-A*68:02. The interacting A chain residues are displayed as red ball and stick, interacting B chain residues are displayed as cyan ball and stick, hydrogen bonds are displayed as green dotted lines, alkyl/pi-alkyl bonds are displayed as pink dotted lines and carbon-hydrogen bonds are displayed as white dotted lines.

**Figure 5:**
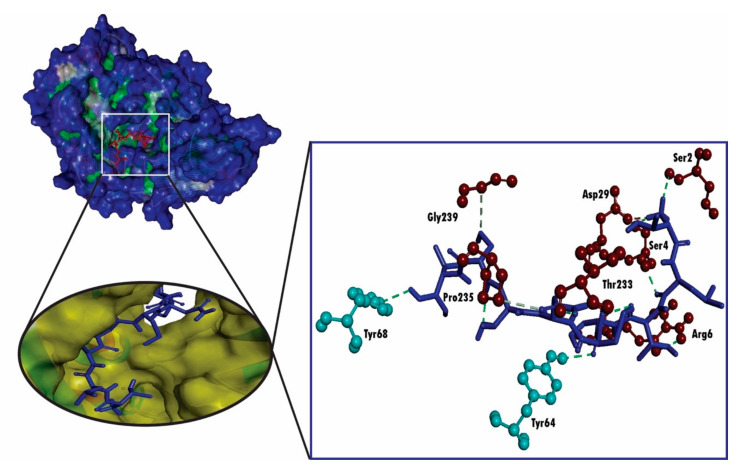
3D representation of molecular docking studies representing the binding affinity of the 9-mer peptide from envelope glycoprotein gp160 form HIV type 1 (positive control) to the groove of the HLA-A*68:02. The interacting A chain residues are displayed as red ball and stick, interacting B chain residues are displayed as cyan ball and stick, hydrogen bonds are displayed as green dotted lines, alkyl/pi-alkyl bonds are displayed as pink dotted lines and salt bridges are displayed as gold dotted lines.

We found that our predicted epitope NTASWFTAL interacted with HLA-A*68:02 with strong binding affinities of -9.4 kcal/mol (Table 4). The selected epitope interacted with Arg6, Ser4, Ser2 and Asp30 residues of chain-A and Lys59, Asp60, Ser58 and Gly30 of chain-B through hydrogen bonding (H-bond), whereas Lys7 residue of chain-B form bonds as a result of sharing electrons (which may happen as a result of charge distribution; Figure 4). Further, for the validation of the docking study, we performed molecular docking analysis between HLA-A*68:02 and the 9-mer peptide bound with the crystal structure of HLA-A*68:02, where the peptide was considered as a positive control. Conversely, the molecular docking analysis between the positive control and HLA-A*68:02 showed less binding affinities than the predicted epitope, where the positive control exhibited a docking score of −8.2 kcal/mol (Table 4). Although the positive control formed six hydrogen bonds, the formed hydrogen bond was less than NTASWFTAL (Figure 5). In addition, a salt bridge was formed between the positive control and Asp29 residue from A chain of HLA-A*68:02.

### 2.9. B-Cell Epitope Prediction

**Figure 6:**
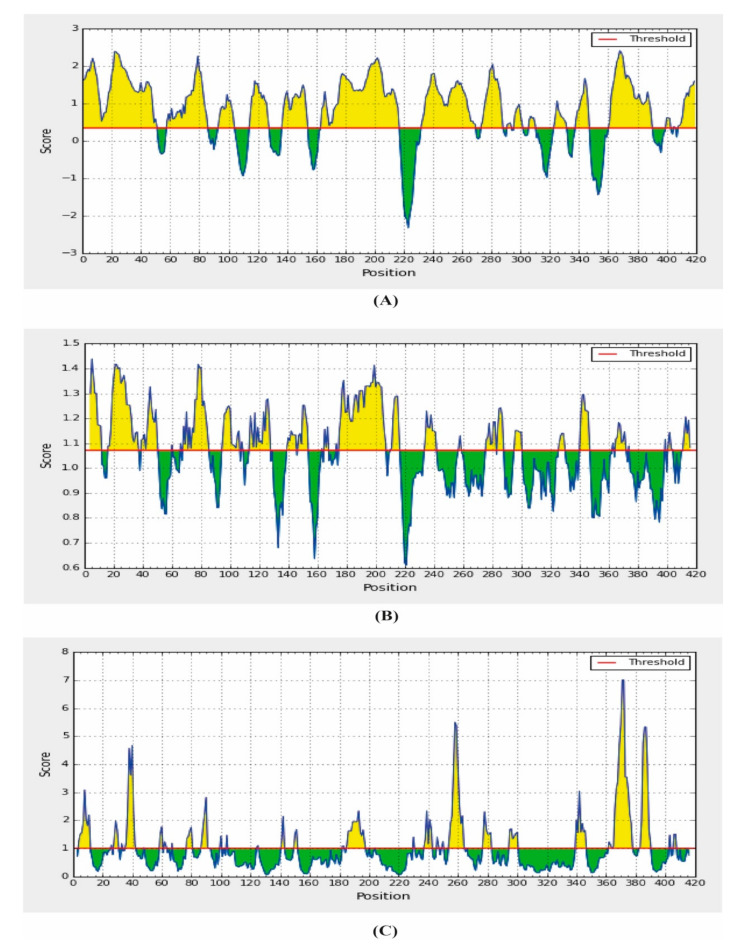
Combined B-cell linear epitope prediction using (**A**) Bepipred linear epitope prediction, (**B**) Chou and Fasman beta-turn prediction and (**C**) Emini surface accessibility prediction methods.

**Figure 7:**
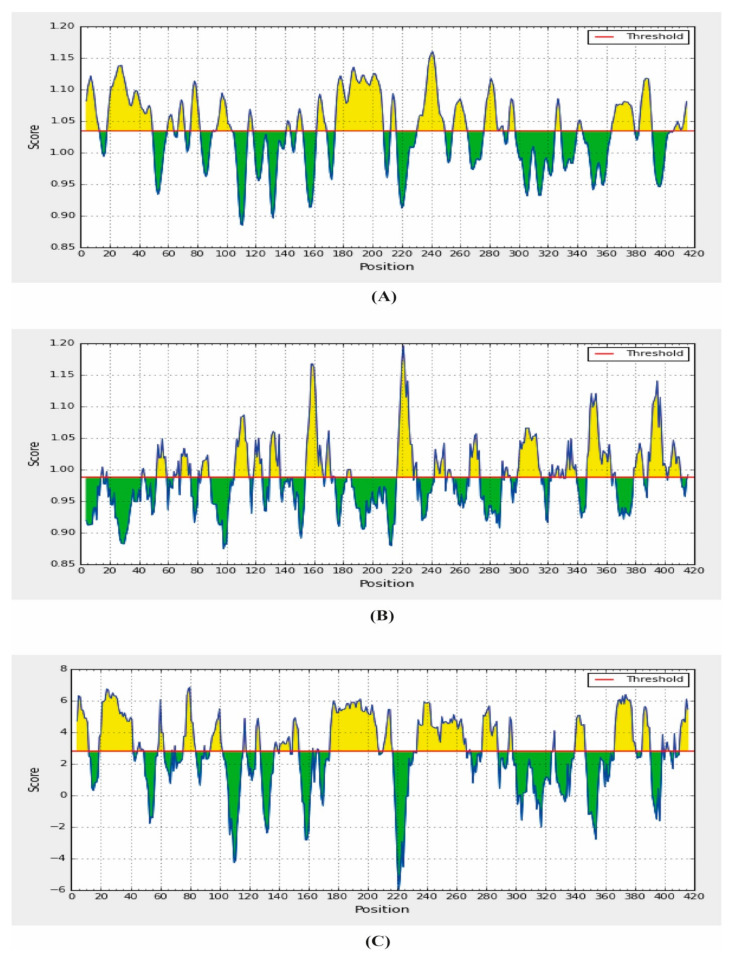
Combined B-cell linear epitope prediction using (**A**) Karplus and Schulz flexibility prediction, (**B**) Kolaskar and Tongaonkar antigenicity and (**C**) Parker hydrophilicity prediction methods.

B-cell epitopes play an important role in the development of epitope-based vaccine and allergic research. A dominant linear B-cell epitope can be used in the autoimmune diseases as the target of neutralizing antibody responses [30]. In addition, they are able to induce an antibody that cross reacts with the parent protein. In this study, using the amino acid scale-based method, we predicted the B-cell epitope identification. Different analysis methods were used for the prediction of the continuous B-cell epitope. The results of the B-cell predictions were shown in Table 5, Table 6 and Table 7, Tables S1 and S2 and Figure 6 and Figure 7.

Firstly, BepiPred linear epitope prediction was used, which is regarded as the best single method for predicting linear B-cell epitopes using a Hidden Markov model. The findings from the BepiPred linear epitope prediction showed maximum score of 2.416 and a minimum score of −0.001, where the average scores were displayed as 0.813 (Table S1).

The β-turns were predicted by the Chaus and Fasman β-turn prediction method. The maximum score was found for the amino residues 2–8 (Figure 6) and the minimum score was attributed for amino acid residues 218–224 (Figure 6).

For antigenicity prediction, the Kolaskar and Tongaonkar antigenicity prediction methods were implied. The method evaluates the antigenicity based on the physicochemical properties of amino acids and their abundances in experimentally known epitopes. The average antigenic propensity of our SARS-CoV-2 N protein was 0.988 with a maximum of 1.197 and a minimum of 0.874 (Figure 7). In addition, the average flexibility of 1.035 and a minimum of 0.874 were predicted by the Karplus and Schulz flexibility prediction method. The residues from 238 to 244 were found to be the most flexible with the highest score of 1.161. The Parker hydrophilicity prediction tool predicts the hydrophilicity of the SARS-CoV-2 N protein with an average score of 2.80, a minimum of 0.874 and the region from amino acid residues 77–83 have shown the maximum score, where the maximum value was 7.006 (Figure 7).

For predicting the surface ability, this study included the Emini surface accessibility prediction method. The average surface accessibility was 1.0 and a minimum 0.050 (Figure 6).

## 3. Discussion

As of yet, it has been reported that the reproduction rate of SARS-CoV-2 is greater than SARS and MERS and the symptoms of the COVID-19 infection include fever with more than 38 °C body temperature along with alveolar edema, leading to difficulty in breathing, whereas mild symptoms perhaps not engender a high fever [31]. Surprisingly, with a high fatality rate, the severity of the infection was found to be more than the infection caused by both SARS and MERS, with multiple organ damage, which was reported not long ago [32].

At present, researchers are examining repurposed compounds from other viral infections to treat SARS-CoV-2. For example, both lopinavir and ritonavir are HIV protease inhibitors but in a lopinavir–ritonavir clinical trial report, the treatment benefit derived was dubious [33]. From recovering patients, several convalescent immunoglobulins are derived, which is currently investigated as a potential treatment for the disease [34]. As there have been no approved treatments for COVID-19 that exists until now, but remdesivir has been used in some emergency cases and evidence also showed that convalescent plasma could be used as treatment without severe adverse effects [34,35]. These treatments are the best hope for striving to keep the mortality rate low before vaccines become widely available.

Despite many potential challenges, vaccine development is a crucial factor in modern biotechnology as vaccines are the most important prerequisites for defending the burden of diseases over the world [36].

With the divulgement of sequence-based technology in genomics and proteomics, enough pieces of information are available regarding different eukaryotic and prokaryotic organisms including viruses. Therefore, utilizing various bioinformatics tools, it is possible to design peptide-based vaccines through comprehensibly studying the epitopes and several studies suggested epitope-based vaccines against different diseases including dengue, chikungunya, Saint Louis encephalitis virus [37,38,39]. Although epitope-dependent vaccine design is quite familiar, little research works are done in the case of SARS-CoV-2. Being an RNA virus, SARS-CoV-2 is different from the DNA virus and the rate of mutation is higher than the DNA viruses and according to various research, it can be assumed that the mutations might occur in the N protein [40]. Recently, N proteins of SARS-CoV-2 are regarded as a primary target for vaccine development as its function includes viral replication and directly associated with the infection process, as a consequence related to the pathogenesis of COVID-19 [41]. Previous research works have already established that N proteins of several viruses and SARS are considered as a potential target for the development of vaccines [42,43,44,45]. Moreover, we already mentioned the detrimental role of SARS-CoV-2 in host–cell responses. This aspect led us to conduct in silico experiments for designing a peptide-based vaccine against the novel SARS-CoV-2.

Earlier, it has been thought that vaccine development primarily relies on B-cell immunity, but recent discovery unveiled that T-cell epitopes are more propitious as a result of a more long-lasting immune response mediated by CD8^+^ T-cells and due to the antigenic drift, by which an antibody is not able to respond against an antibody [46]. In this study, focusing on MHC class I potential peptide epitopes, we predicted T-cell and B-cell epitopes, which were able to show immune responses in various ways. Many characteristics including antigenicity, toxicity need to take into consideration for identifying a protein sequence-based epitope into a vaccine candidate and the predicted eight epitopes fulfilled the entire criterion. Toxicity analysis is regarded as an important parameter during design of a peptide sequence into a vaccine candidate. For instance, melittin, a major peptide of bee venom, is a promising candidate for cancer therapy, but due to its toxicity, its applicability has met with critical challenges [47]. In the current study, only five potent epitopes have been predicted from the NetCTL 1.2 server and the epitopes were further taken for the progressive analysis. Besides, all peptides except SSPDDQIGY were able to interact with the MHC class I alleles, and NTASWFTAL interacted with the most MHC class I alleles. Amongst them, HLA-A*68:02 possessed the highest probable score. Further, the conservancy of the epitopes, which was predicted by the IEDB conservancy analysis tool delineated that all of our predicted epitopes had the maximum identity of 100%. Apart from this, a computational study unraveled that the targeted epitope NTASWFTAL showed conservancy along with several epitopes from SARS-CoV-2 [48]. Previously, NTASWFTAL has been used in order to determine the ability to elicit the SARS-CoV immune response [49]. Furthermore, a previous study has already demonstrated that NTASWFTAL interacted with most of the HLA supertypes, including, HLA-A*01:01, HLA-A*02:01, HLA-A*03:01, HLA-A*24:02, HLA-A*26:01, HLA-B*07:02, HLA-B*08:01, HLA-B*27:05, HLA-B*39:01, HLA-B*40:01, HLA-B*58:01 and HLA-B*15:01 [50]. The amino sequence GLPNNTASWFTALTQHGK of SARS-COV-2 N protein also demonstrated the characteristics of the B-cell epitope, which includes the targeted epitope NTASWFTAL [51]. Therefore, we took the epitope NTASWFTAL for further analysis due to its maximum interaction with MHC class I alleles and the highest conservancy.

Generally, allergy is considered as an overreaction of the immune system to a previously captured, harmless, normal protein in nature. True allergic reactions to vaccines are rare; however, their identification is crucial because they can be detrimental to the body [52]. Occasionally, the vaccine itself causes hypersensitivity due to the toxoids present in it. Hence, allergenicity is regarded as one of the most noteworthy obstacles in vaccine development. Importantly, T-cells not CD4^+^ T-cells are involved in an allergic reaction and an allergic reaction is stimulated by type 2 T helper cell along with immunoglobulin E [53]. In this experiment, we assessed the allergenicity using AllerTop 2.0, which is well recognized for its high sensitivity, and able to identify structurally diverse allergens in comparison with the known allergens. AllerTop predicted our selected epitope as non-allergen.

It has been proposed that the T-cell epitopes bind with the MHC molecules and MHC class I molecules generally presented short peptides that are 8–11 amino acid long, whereas MHC class II molecules present longer peptides with 13–17 amino acid residues [54]. In this experiment, we determined the binding (presence of the antigen on the surface) affinity of the predicted epitope using molecular docking analysis and demonstrated that NTASWFTAL interacted with HLA-A*68:02 and found a binding affinity of −9.4 kcal/mol, which depicted a greater interaction with the epitope and the HLA molecule as the more negative energy implied to more binding affinity [55]. In addition, our predicted epitope delineated greater binding affinities to HLA-A*68:02 than its native ligand. Importantly, a study from Zhang reported the highest binding affinity of NTASWFTAL towards the HLA-A2/A0201-restricted T-cell epitopes [56]. The results from the molecular docking studies in the current study also revealed that epitope NTASWFTAL formed H-bond with both chain-A and chain-B of the HLA molecule and attractive charges were also responsible for the binding.

Another factor that is considered as the most prominent one during the process of vaccine development is population coverage, as the distribution of HLA varies according to ethnicity and geographical region. Although after implementation of several clinical studies, genetic variability on a global scale could have an effect on the significant application of the vaccine candidates in humans [57]. Our experiment showed that the epitope NTASWFTAL covered almost all regions of the world, where the highest coverage was observed in East Asia, where COVID-19 was first reported. Interestingly, our findings indicated that our predicted epitope specifically binds with the widespread HLA molecules and the vaccine will be easily employed.

Importantly, the accurate prediction of T-cell epitopes along with B-cell epitopes is a crucial challenge for the immunoinformatics study and it is unlikely that different HLAs are expressed at different frequencies amongst the ethnic groups. However, substantial research in several in silico markers including the matrix-based profile, and regular expressions in the immunoinformatics study provide a cogent way for prediction of several immunobiological phenomena, for instance, the prediction of subcellular localization (SCL) of a protein is identified by several computational tools. Similarly, T-cell epitope identification has been undergone by implying numerous computational methods and various research areas, including cancer therapy and other infections, T-cell epitope identification is presently apparent [58,59,60]. Additionally, experimental methods established for the calculation of the binding interaction between MHC molecules and an antigenic protein is complicated and time-consuming. Hence, several computational tools have been introduced concerning simulation of the experimental methods, and the methods of the MHC binder prediction are based on motifs, quantitative matrices (QMs), ab initio prediction, machine-learning techniques, DiscoTope, etc. [61]. Several algorithms including PePSSI (peptide–MHC prediction of structure through solvated interfaces) and PREDEP (prediction of MHC class I epitopes) are implemented for the structural prediction and side-chain orientation of the binding proteins. In the current study, the prediction of MHC-I binding with T-cell antigenic peptides from the SARS-CoV-2 N protein sequences was done through the SMM algorithm, which incorporates proteasomal cleavage, TAP transport and MHC class I affinity into the final output and recent studies suggested that SMM is more established than other algorithms such as EpiJen and MAPPP [62,63,64].

Recently, other research works have suggested vaccine design from antigenic protein sequences of SARS-CoV-2 through utilizing in silico immunoinformatics-based methodologies. A study from Lee et al. reported a comprehensive list of antigenic peptides for vaccine development against SARS-CoV-2 [65]. However, the findings of the research work represented that the N protein patterns retained from SARS-CoV-2 were unable to interact with HLA alleles. Several other studies also delineated the high binding affinity of predicted epitopes towards HLA-A*24:02 and HLA-A*02:01 alleles respectively [66,67]. Conversely, in the current research work, our predicted epitope NTASWFTAL exhibited greater affinity towards HLA-A*68:02, predicted by NetCTL 1.2 server. Besides, molecular docking simulation unveiled the greater interaction between the predicted epitope and HLA-A*68:02 molecules. Moreover, our current study is in alignment with previous research work, which depicted peptide-based sequence against the S protein of the human coronavirus [36]. However, we cannot rule out the role of MHC class II peptides during the design of the epitope-based vaccine, as it plays a phenomenal role in humoral immunity through helping B-cells.

In addition, the B-cell epitope provides a strong immune response without causing any adverse effects. Generally, B-cell epitopes are either linear (continuous) or conformational (non- continuous) [68]. Importantly, flexible regions are observed in several crucial parts of a protein, including binding sites, catalytic sites, proteolytic cleavage susceptible sites, allosteric sites and most importantly the antigenic part of a protein sequence. Flexibility analysis is one of the major concerns for the identification of the surface residues forming a protein, which is further demonstrated as potential continuous epitopes [69]. For vaccine development, it would be crucial for predicting the antigenic region. In addition, hydrophilic amino acid residues are major determinants of antigenic features of a protein sequence, as the point highest hydrophilicity is located in or adjacent to an antigenic portion of the protein [70]. In this experiment, we also calculated the linear B-cell epitope prediction. It has been documented that peptide vaccines that are able to demonstrate immune responses against foreign particles contain peptides that are comprised of linear B cell epitopes [71]. B cell epitopes carry specific antigens that bind to the B lymphocytes, as a result they are recognized as potential antigenic determinants and are crucial for vaccine design [72]. In addition, B cell epitopes elicited a stronger immune response, but no side effects were observed. Recently, Grifoni et al. predicted B cell epitopes by utilizing the structural proteins of SARS-CoV and SARS-CoV-2 [73]. The Grifoni study predicted the identity of three peptide sequences from 42–62, 153–172 and 355–401 amino acid residues having an identity ≥ 90% [73]. In the current experiment, by using several tools from IEDB database, we predicted several B-cell epitopes from the SARS-CoV-2 N protein. As a consequence, our study predicted several B cell epitopes that were in line with those identified by Grifoni et al. (Table S2). Additionally, one of the predicted B-cell epitope from amino acid residues 154–166 was in agreement with the study from Amrun et al. (Table S2) [74]. Moreover, several studies have reported the characterization of B-cell epitopes from the N protein of many viruses from humans and animals [23,75,76,77].

Recently, immunoinformatics-aided vaccine design has received experimental validation, which targeted multi-epitope protein clusters from *Mycobacterium tuberculosis* that interacted with HLA class I and II molecules and their prediction was experimentally validated through in vitro studies [78]. On the other hand, our study was more specific than some similar studies, for example, a study from Khan et al. had selected MHC-I alleles for which the epitopes representing higher affinity (IC_50_ < 500 nm), but in our study, we showed that epitopes for MHC I alleles showing higher affinity (IC_50_ < 200 nm), as peptides with minimum IC_50_ values, exhibited greater inhibition [79,80]. In addition, we assessed immunogenicity, allergenicity and toxicity of the selected epitopes. Moreover, B-cell epitopes can pave the way for experimental epitope mapping and also crucial concerning the interpretation of results from several experiments, including ELISA, radioimmunoassay and Western blotting.

Of course, we understood that this research work does not claim to be exhaustive and all-inclusive as it is true that in silico works have its advantages and limitations. However, recently immunoinformatics is regarded as a new branch of computational biology techniques and is effective in the quest of new immunotherapeutics, amalgamating bioinformatics techniques to figure out several unique problems of vaccinology and immunology [81]. Epitope prediction can be regarded as a high parameter in immunoinformatics investigation, and immunoinformatics calculations are considered as the high frontier to develop effective vaccines true of the practical value. However, the experimental validations of the underlying approaches are required to establish a predicted epitope into a vaccine candidate. The accuracy of the predicted computational analysis should be corroborated by more accessible and robust laboratory experiments.

## 4. Materials and Methods

### 4.1. Protein Sequence Retrieval

The SARS-CoV-2 N protein sequence was extracted from the NCBI (National Center for Biotechnology Information) (Bethesda, MD, USA) protein database (Accession no.: QIC53221.1, GI: 1811294683) in the FASTA format.

### 4.2. Sequence Analysis

The understanding of the features, function, structure and evaluation is mainly based on the process of sequence analysis, which depicts the process of subjecting DNA, RNA or peptide sequences to wide ranges of analytical methods. We employed NCBI BLAST (Basic Local Alignment Search Tool) [82] that screens homologous sequences from its database and selects those sequences that are more similar to our SARS-CoV-2 N protein; we also performed multiple sequence alignment (MSA) using the ClustalW (Conway Institute, UCD, Dublin, Ireland) web server with default settings, and a phylogenetic tree was assembled using MEGA6 software [82,83,84].

### 4.3. Protein Antigenicity and Toxicity Prediction

To determine the potent antigenic protein of the SARS-CoV-2 N protein, we used the online server VaxiJen v2.0, with a default threshold value [85]. All the antigenic proteins of SARS-CoV-2 N protein with their respective scores were obtained then sorted in Notepad++. A single antigenic protein with maximum antigenicity scores was selected for further evaluation. The toxicity of epitopes was analyzed using the ToxinPred web server [86].

### 4.4. Protein Secondary and Tertiary Structure Prediction

The secondary structure of the SARS-CoV-2 N protein was predicted by using the SOPMA tool (Institute of Biology and Protein Chemistry, Lyon, France), which correctly predicts 69.5% of amino acids for a three-state description of the secondary structure (α-helix, β-sheet and coil) in a whole database [27]. Additionally, we predicted the 3D structure of the protein using Robetta (University of Washington, Seattle, WA, USA) server, which provides automated tools for prediction and analysis of the tertiary structure of the protein [28]. The model was validated using PROCHECK and PROSA web servers [87,88]. In addition, the 3D crystal structure of SARS-CoV-2 N protein (PDB ID: 6M3M) was downloaded from the Protein Data Bank (PDB) database for comparing the modeled 3D structure of the SARS-CoV-2 N protein.

### 4.5. T-Cell Epitope Prediction

#### CD8^+^ T-Cell Epitope Prediction

For the de novo prediction of the T-cell epitope, NetCTL 1.2 server (DTU Health Tech, Kongens Lyngby, Denmark) was used in this experiment, using a 0.95 threshold to maintain the sensitivity and specificity of 0.90 and 0.95, respectively. The tool expands the prediction for 12 MHC-I supertypes and integrates the prediction of peptide MHC-I binding and proteasomal C-terminal cleavage with TAP transport efficiency. These predictions were performed by an artificial neural network, weighted TAP transport efficiency matrix and a combined algorithm for MHC-I binding and proteasomal cleavage efficiency was then used to determine the overall scores and translated into sensitivity/specificity. Based on this overall score, five best peptides (epitopes) were selected for further evaluation.

For the prediction of peptides binding to MHC-I, we used a tool from the Immune Epitope Database (IEDB) (National Institute of Allergy and Infectious Diseases, Bethesda, MD, USA) and calculate IC_50_ values for peptides binding to specific MHC-I molecules [89]. For the binding analysis, all the frequently used alleles were selected with a word length of nine residues and binding affinity <200 nm for further analysis. Another tool (named as MHC-NP) provided by the IEDB server was used to assess the probability that a given peptide was naturally processed and bound to a given MHC molecule [90].

### 4.6. Epitope Conservancy and Immunogenicity Prediction

The degree of similarity between the epitope and the target (i.e., given) sequence was elucidated by epitope conservancy. This property of the epitope gave us the promise of its availability in a range of different strains. Hence for the analysis of the epitope conservancy, the web-based tool from IEDB analysis resources was used [91]. Immunogenicity prediction can uncover the degree of influence (or efficiency) of the respective epitope to produce an immunogenic response. The T-cell class I pMHC immunogenicity predictor at IEDB, which uses amino acid properties as well as their position within the peptide to predict the immunogenicity of a class I peptide MHC (pMHC) complex [92].

### 4.7. Prediction of Population Coverage and Allergenicity Assessment

The population coverage tool from IEDB was applied to determine the population coverage for every single epitope by selecting HLA alleles of the corresponding epitope.

Allergenicity of the predicted epitope was calculated using AllerTop v2.0 (Medical University, Sofia, Bulgaria) [29], which is an alignment-free server, used for in silico based allergenicity prediction of a protein-based on its physiochemical properties.

### 4.8. HLA and Epitope Interaction Analysis Using Molecular Docking Studies

#### 4.8.1. Epitope Model Generation

The 3D structures of the selected epitopes were predicted by PEP-FOLD, a web-based server [93]. For each sequence, the server predicted five probable structures. The energy of each structure was determined by SWISS-PDB VIEWER and the structure with the lowest energy was chosen for further analysis [94].

#### 4.8.2. Retrieval of the HLA Allele Molecule

The three-dimensional structure of the HLA-A*68:02 (PDB ID: 4I48) was retrieved from Protein Data Bank (RCSB-PDB).

#### 4.8.3. Molecular Docking Analysis

Molecular docking analysis was performed using AutoDock vina (Scripps Research, La Jolla, CA, USA) in PyRx 0.8, by considering the HLA-A*68:02 molecule as the receptor protein and identified epitopes as the ligand molecule [95]. Firstly, we used the protein preparation wizard of UCSF Chimera (Version 1.11.2) to prepare the protein for docking analysis by deleting the attached ligand, adding hydrogens and Gasteiger–Marsili charges [96,97]. The prepared file was then added to the AutoDock wizard of PyRx 0.8 and converted into the pdbqt format. The energy form of the ligand was minimized and converted to the pdbqt format by OpenBabel [98]. The parameters used for the docking simulation were set to the default. The size of the grid box in AutoDock Vina was kept at 50.183 Å × 50.183 Å × 50.183 Å respectively, for X, Y and Z-axis. AutoDock Vina was implemented via the shell script offered by AutoDock Vina developers [99]. Docking results were observed by the negative score in kcal/mol, as the binding affinity of ligands are depicted in negative energies [100,101]. In addition, for validation of the docking approach, we selected 9-mer peptide from the envelope glycoprotein gp160 from human immunodeficiency virus (HIV) type 1 attached with the crystal structure of HLA-A*68:02 as a positive control and performed molecular docking analysis using the aforementioned similar parameters.

### 4.9. B-Cell Epitope Identification

The prediction of B-cell epitopes was performed to find the potential antigen that assures humoral immunity. To detect the B-cell epitope, various tools from IEDB were used to identify the B-cell antigenicity, together with the Emini surface accessibility prediction, Kolaskar and Tongaonkar antigenicity scale, Karplus and Schulz flexibility prediction and Bepipred linear epitope prediction analysis and since antigenic parts of a protein belonging to the beta-turn regions, the Chou and Fasman beta-turn prediction tool was also used [102,103,104,105,106,107].

## 5. Conclusions

The advancement in immunoinformatics has now emerged as a potential field for the prediction of epitope-based vaccines. As viruses can delineate both T-cell and humoral immunity, our predicted epitope might suggest enhancing the immunity against SARS-CoV-2. The assumption is based on the basic principles of immunity, which confers the attachment of the virus with the host cell, evoking immune responses and transfers the information to a broad spectrum of T cells and B cells. Our investigated epitopes mimicked the interaction to CD8^+^ cells antigen presentation using computational approaches. However, our study was an introductory design to predict epitope-based vaccine against SARS-CoV-2 and we hope that this predicted epitope would assist the further laboratory analysis for designing and predicting novel candidates against COVID-19.

## Figures and Tables

**Table 1:** Toxicity prediction of selected epitopes.

Epitopes	Toxicity Prediction	SVM Score	Hydrophobicity	Hydrophilicity	Molecular Weight
LSPRWYFYY	Non-Toxin	−1.08	−0.06	−1.26	1294.59
GTTLPKGFY	Non-Toxin	−1.13	−0.01	−0.49	983.26
DLSPRWYFY	Non-Toxin	−1.18	−0.14	−0.67	1246.5
SSPDDQIGY	Non-Toxin	−0.33	−0.2	0.3	981.1
LLNKHIDAY	Non-Toxin	−0.81	−0.09	−0.28	1086.39
GTDYKHWPQ	Non-Toxin	−0.29	−0.29	−0.04	1131.34
SPDDQIGYY	Non-Toxin	−0.54	−0.17	0.01	1057.2
NTASWFTAL	Non-Toxin	−1	0.08	-1	1010.23

**Table 2:** The potential CD8^+^ T-cell epitopes along with their interacting MHC class I alleles and total processing score, epitopes conservancy_hits and pMHC-I immunogenicity score.

Epitopes	NetCTL Combined Score	Epitope Conservancy Hit (MAX. Identity %)	MHC-I Interaction with an Affinity of IC_50_ < 200 and the Total Score (Proteasome Score, TAP Score, MHC-I Score, Processing Score)	pMHC-I Immunogenicity Score
LSPRWYFYY	2.3408	100	HLA-A*29:02 (1.32), HLA-A*30:02 (0.8), HLA-A*01:01 (0.66), HLA-C*16:01 (0.26)	0.35734
NTASWFTAL	0.9521	100	HLA-A*68:02 (1.11), HLA-C*16:01 (0.18), HLA-C*03:03 (0.12), HLA-C*03:04 (0.12), HLA-C*12:03 (0.10), HLA-A*02:06 (0.04), HLA-C*03:02 (−0.07), HLA-A*26:01 (−0.13), HLA-C*14:02 (−0.34)	0.22775
DLSPRWYFY	1.4994	100	HLA-A*29:02 (0.99)	0.25933
SPDDQIGYY	1.1404	100	HLA-B*35:01 (0.52)	0.06844
SSPDDQIGY	0.6895	100		0.0634

**Table 3:** Analysis of the population coverage for the proposed epitope against SARS-CoV-2.

Population	Coverage (%) ^a^	Average Hit ^b^	PC90 ^c^
Central Africa	35.31	0.40	0.15
East Africa	39.25	0.45	0.16
East Asia	57.56	0.71	0.24
Europe	42.95	0.50	0.18
North Africa	42.15	0.49	0.17
North America	45.32	0.53	0.18
Northeast Asia	48.11	0.55	0.19
Oceania	31.43	0.34	0.15
South Africa	33.91	0.38	0.15
South America	38.66	0.44	0.16
South Asia	36.53	0.41	0.16
Southeast Asia	49.45	0.57	0.20
Southwest Asia	28.53	0.32	0.14
West Africa	56.22	0.67	0.23
West Indies	12.89	0.13	0.11

Notes: ^a^ Projected population coverage. ^b^ Average number of epitope hits/HLA combinations recognized by the population. ^c^ Minimum number of epitope hits/HLA combinations recognized by 90% of the population.

**Table 4:** Results of the molecular docking analysis amongst HLA-A*68:02 and the predicted epitope, NTASWFTAL, and 9-mer peptide from envelope glycoprotein gp160 from HIV type 1 (positive control).

Epitopes	Docking Score (kcal/mol)
NTASWFTAL	−9.4
Positive Control	−8.2

**Table 5:** List of predicted B cell epitopes from BepiPred linear epitope prediction analysis.

Start	End	Peptide	Length
361	390	KTFPPTEPKKDKKKKADETQALPQRQKKQQ	30
338	347	KLDDKDPNFK	10
323	331	EVTPSGTWL	9
273	287	AFGRRGPEQTQGNFG	15
232	269	SKMSGKGQQQQGQTVTKKSAAEASKKPRQKRTATKAYN	38
164	216	GTTLPKGFYAEGSRGGSQASSRSSSRSRNSSRNSTPGSSRGTSPARMAGNGGD	53
137	154	GALNTPKDHIGTRNPANN	18
115	127	TGPEAGLPYGANK	13
93	104	RIRGGDGKMKDL	12
58	85	QHGKEDLKFPRGQGVPINTNSSPDDQIG	28
1	51	MSDNGPQNQRNAPRITFGGPSDSTGSNQNGERSGARSKQRRPQGLPNNTAS	51

**Table 6:** List of predicted B-cell epitopes from Kolaskar and Tongaonkar antigenicity prediction method.

Start	End	Peptide	Length
52	59	WFTALTQH	8
69	75	GQGVPIN	7
83	89	QIGYYRR	7
106	115	PRWYFYYLGT	10
130	136	IIWVATE	7
154	166	NAAIVLQLPQGTT	13
217	227	AALALLLLDRL	11
243	249	GQTVTKK	7
267	273	AYNVTQA	7
299	315	KHWPQIAQFAPSASAFF	17
333	339	YTGAIKL	7
347	363	KDQVILLNKHIDAYKTF	17
379	385	TQALPQR	7
389	401	QQTVTLLPAADLD	13
403	411	FSKQLQQSM	9

**Table 7:** List of predicted B-cell epitopes from Emini surface accessibility prediction method.

Start	End	Peptide	Length
4	11	NGPQNQRN	8
36	42	RSKQRRP	7
185	197	RSSSRSRNSSRNS	13
254	264	ASKKPRQKRTA	11
277	282	RGPEQT	6
295	300	GTDYKH	6
340	346	DDKDPNF	7
365	377	PTEPKKDKKKKAD	13
384	390	QRQKKQQ	7

## Supplementary Materials

The following are available online. Data S1: Multiple sequence alignment of SARS-CoV-2 nucleocapsid protein; Figure S1: Evolutionary divergence analysis of available N proteins of different strains; results are represented in a phylogenetic tree; Figure S2: (A) Ramachandran plot analysis for model 1, (B) Ramachandran plot analysis for model 2, (C) Ramachandran plot analysis for model 3, (D) Ramachandran plot analysis for model 5, (E) Ramachandran plot analysis for the crystal structure of SARS-CoV-2 N protein (PDB ID; 6M3M), (F) Z-score for model 1, (G) Z-score for model 2, (H) Z-score for model 3, (I) Z-score for model 5 and (J) Z-score for the crystal structure of SARS-CoV-2 N protein (PDB ID; 6M3M); Figure S3: Population coverage based on MHC restriction data for (A) Central Africa, (B) East Africa, (C) East Asia and (D) North Africa—using the Immune Epitope Database analysis resource; Figure S4: Population coverage based on MHC restriction data for (A) North Africa, (B) North America, (C) Northeast Asia and (D) Oceania—using the Immune Epitope Database analysis resource; Figure S5: Population coverage based on MHC restriction data for (A) South Africa, (B) South America, (C) South Asia and (D) Southeast Asia—using the Immune Epitope Database analysis resource; Figure S6: Population coverage based on MHC restriction data for (A) Southwest Asia, (B) West Africa and (C) West Indies—using the Immune Epitope Database analysis resource. Table S1: Scores from the combined B-cell linear epitope prediction; Table S2. Predicted B-cell epitopes linear with the previous studies from Grifoni et al. and Amrun et al.
