# Supplementary material for: Epitope-Based Immunoinformatics Approach on Nucleocapsid Protein of Severe Acute Respiratory Syndrome-Coronavirus-2
Source: Molecules. 2020 Nov 2;25(21):5088. doi: 10.3390/molecules25215088 (PMC7663370; doi:10.3390/molecules25215088)
Supplement: Supplementary file 1 [file molecules-25-05088-s001.pdf]

Supplementary Materials

Article

Epitope-Based Immunoinformatics Approach on Nucleocapsid Protein of Severe Acute Respiratory Syndrome-Coronavirus-2

Ahmed Rakib [1], Saad Ahmed Sami [1], Md. Ashiqul Islam [1,2], Shahriar Ahmed [1], Farhana Binta Faiz [1], Bibi Humayra Khanam [1], Kay Kay Shain Marma [1], Maksuda Rahman [1], Mir Muhammad Nasir Uddin [1], Firzan Nainu [3], Talha Bin Emran [4,*] and Jesus Simal-Gandara [5,*]

[1] Department of Pharmacy, Faculty of Biological Sciences, University of Chittagong, Chittagong-4331, Bangladesh; rakib.pharmacy.cu@gmail.com (A.R.); s.a.sami18pharm@gmail.com (S.A.S.); ashiqulanik@gmail.com (M.A.I.); shahriarahmed65@gmail.com (S.A.); farhanafaiz037@gmail.com (F.B.F.); humayra.Khanam@cu.ac.bd (B.H.K.); kkshainapu@gmail.com (K.K.S.M.); maksudarahman777@gmail.com (M.R.); nasirmir@cu.ac.bd (M.M.N.U.)

[2] Department of Pharmacy, Mawlana Bhashani Science & Technology University, Santosh, Tangail-1902, Bangladesh; ashiqulanik@gmail.com (M.A.I.)

[3] Faculty of Pharmacy, Hasanuddin University, Tamalanrea, Kota Makassar, Sulawesi Selatan 90245, Indonesia; firzannainu@unhas.ac.id (F.N.)

[4] Department of Pharmacy, BGC Trust University Bangladesh, Chittagong-4381, Bangladesh

[5] Nutrition and Bromatology Group, Department of Analytical and Food Chemistry, Faculty of Food Science and Technology, University of Vigo–Ourense Campus, E32004 Ourense, Spain

* Correspondence: jsimal@uvigo.es (J.S.-G.); talhabmb@bgctub.ac.bd (T.B.E.)

Tel.: +34-988-387000 (J.S.G.); +88-01819-942214 (T.B.E.)

Table S1. Scores from the combined B-cell linear epitope prediction.

Method	Score		
	Maximum	Average	Minimum
BepiPred Linear Epitope Prediction	2.416	0.813	-0.001
Chou and Fasman Beta-Turn Prediction	1.439	1.070	0.610
Emini Surface Accessibility Prediction	7.006	1.000	0.050
Karplus and Schulz Flexibility Prediction	1.161	1.035	0.885
Kolaskar and Tongaonkar Antigenicity	1.197	0.988	0.874
Parker Hydrophilicity Prediction	6.871	2.800	-5.971

Table S2. Predicted B cell epitopes linear with the previous studies from Grifoni *et al*.,and Amrun *et al*.

No.	Start	End	Peptide	Length
1	361	390	KTFPPTEPKKDKKKKADETQALPQRQKKQQ	30
2	52	59	WFTALTQH	8
3	154	166	NAAIVLQLPQGTT	13
4	347	363	KDQVILLNKHIDAYKTF	17
5	379	385	TQALPQR	7
6	389	401	QQTVTLLPAADLD	13
7	365	377	PTEPKKDKKKKAD	13
8	384	390	QRQKKQQ	7

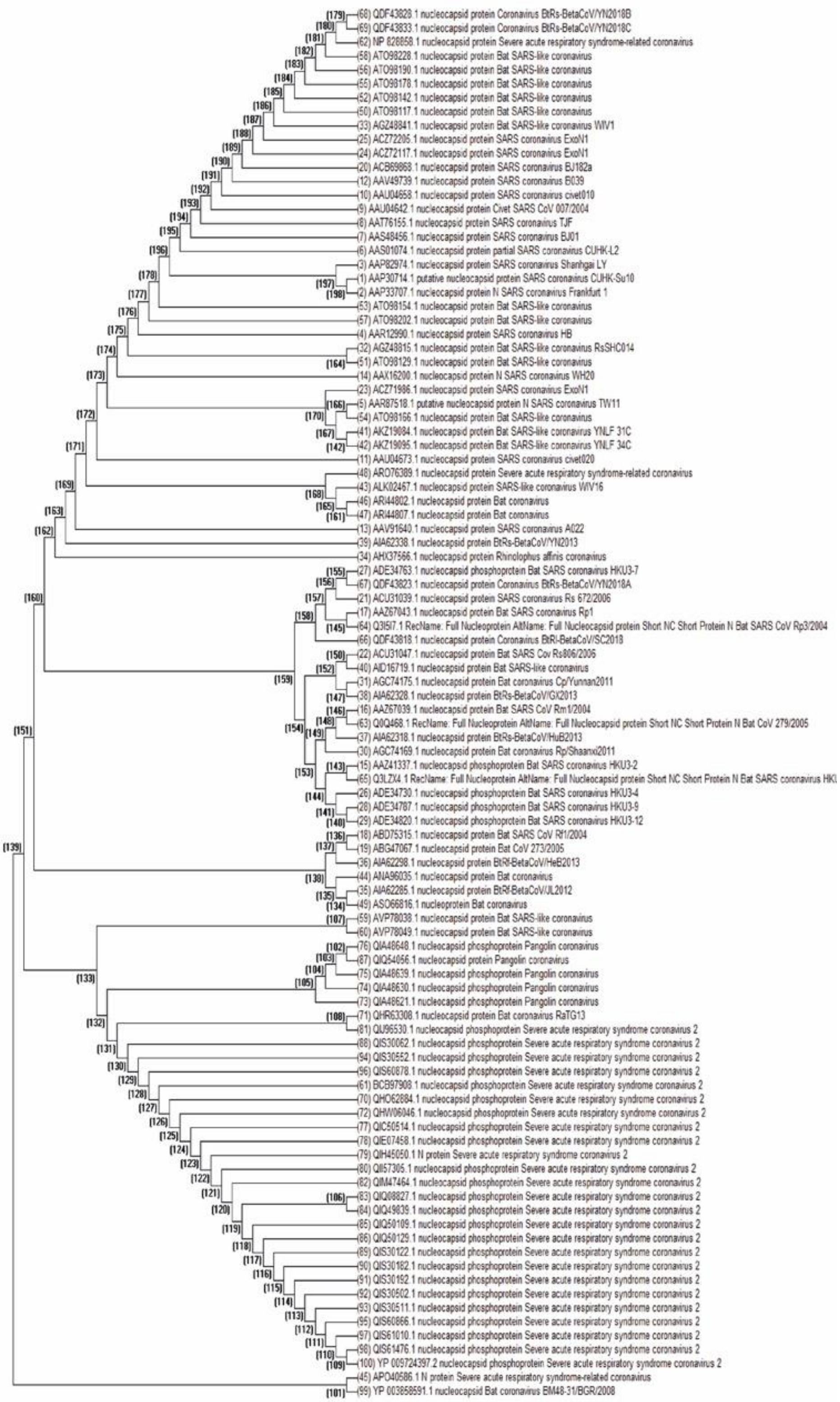

Figure S1: Evolutionary divergence analysis of available N proteins of different strains; results are represented in a phylogenetic tree.

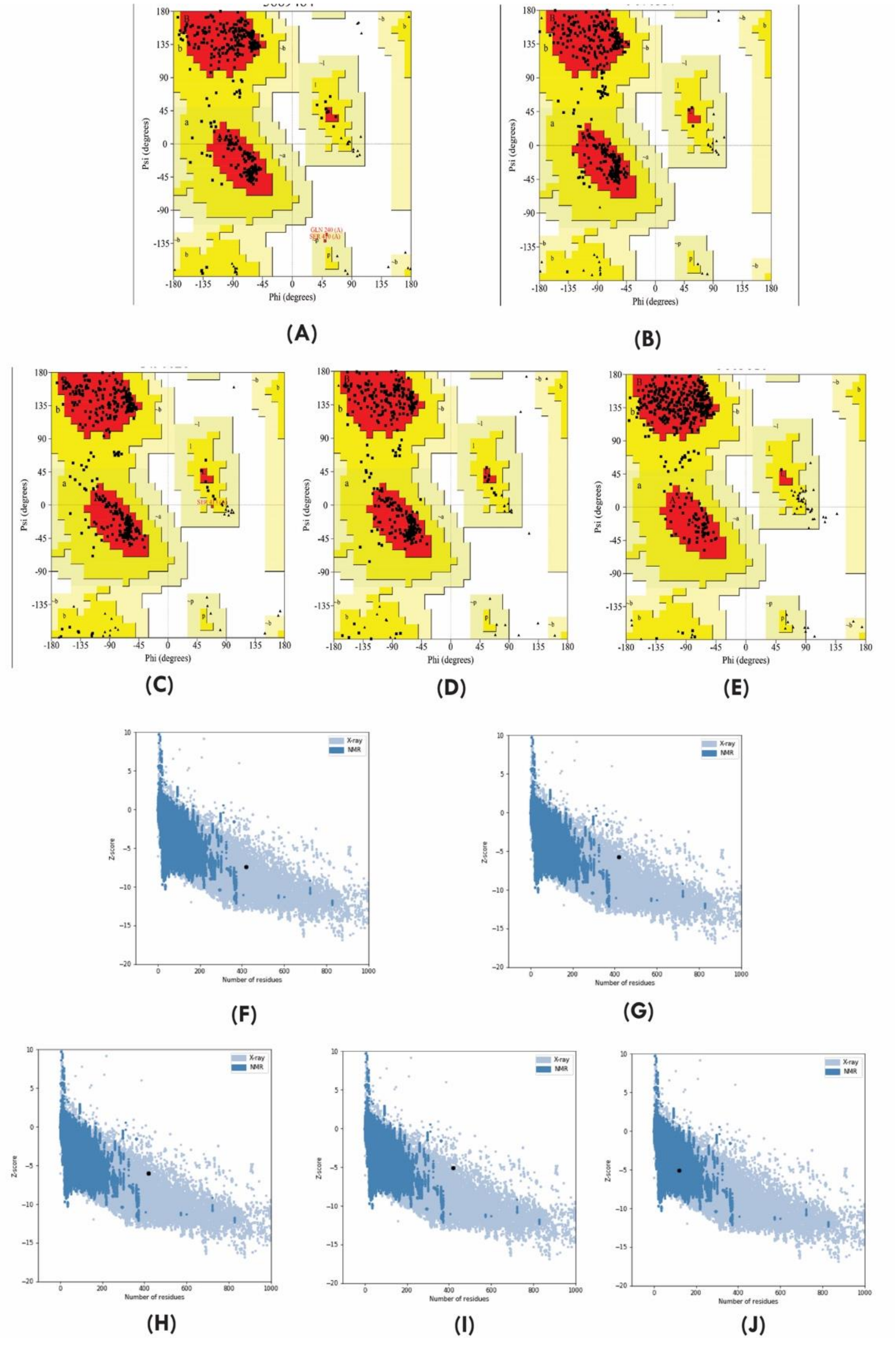

Figure S2: **(A)** Ramachandran plot analysis for model 1; **(B)** Ramachandran plot analysis for model 2; **(C)** Ramachandran plot analysis for model 3; **(D)** Ramachandran plot analysis for model 5; **(E)** Ramachandran plot analysis for the crystal structure of SARS-CoV-2 N protein (PDB ID; 6M3M); **(F)** Z-score for model 1; **(G)** Z-score for model 2; **(H)** Z-score for model 3; **(I)** Z-score for model 5; **(J)** Z-score for the crystal structure of SARS-CoV-2 N protein (PDB ID; 6M3M).

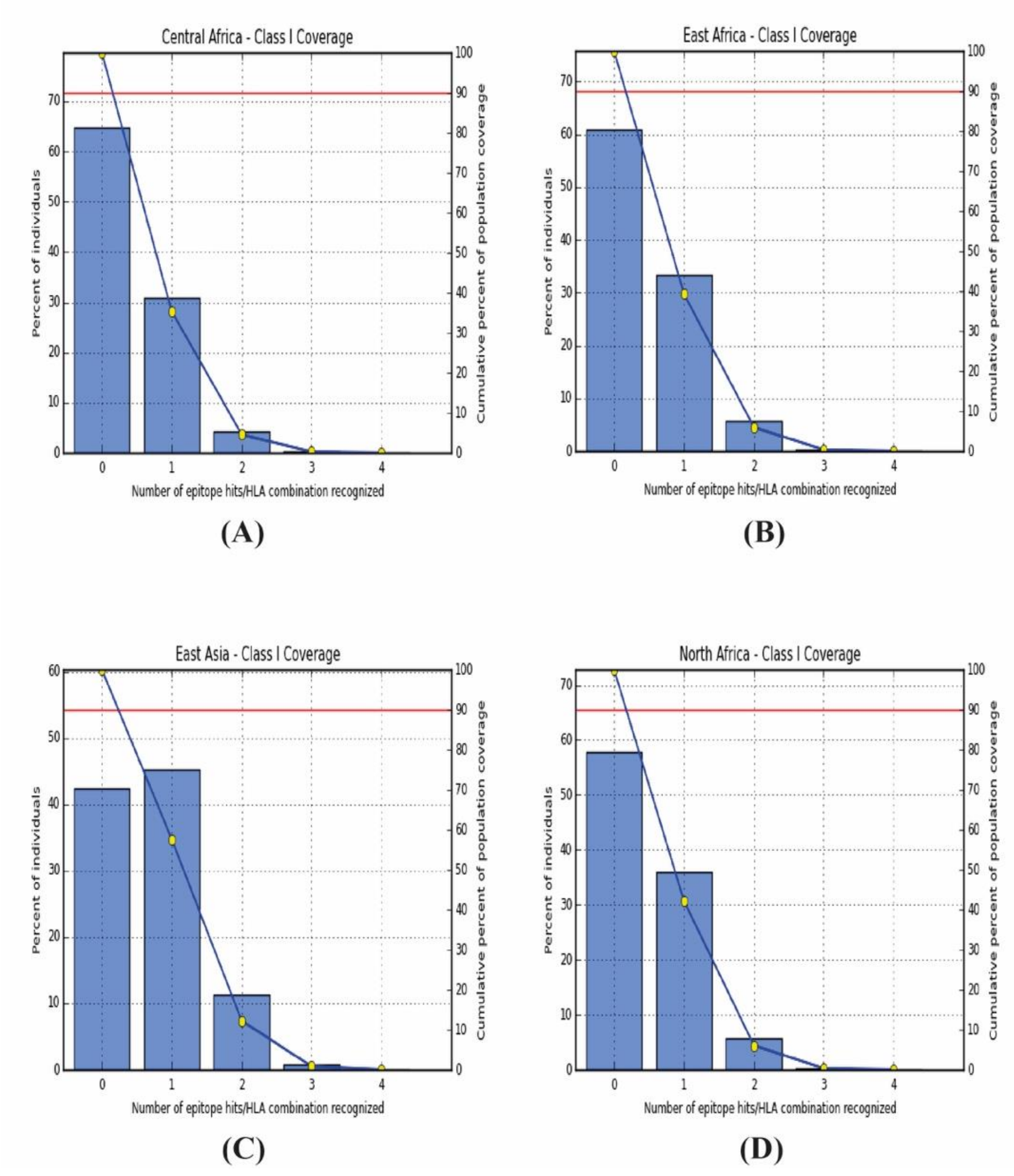

Figure S3: Population coverage based on MHC restriction data for **(A)** Central Africa; **(B)** East Africa; **(C)** East Asia; **(D)** North Africa - using the Immune Epitope Database analysis resource.

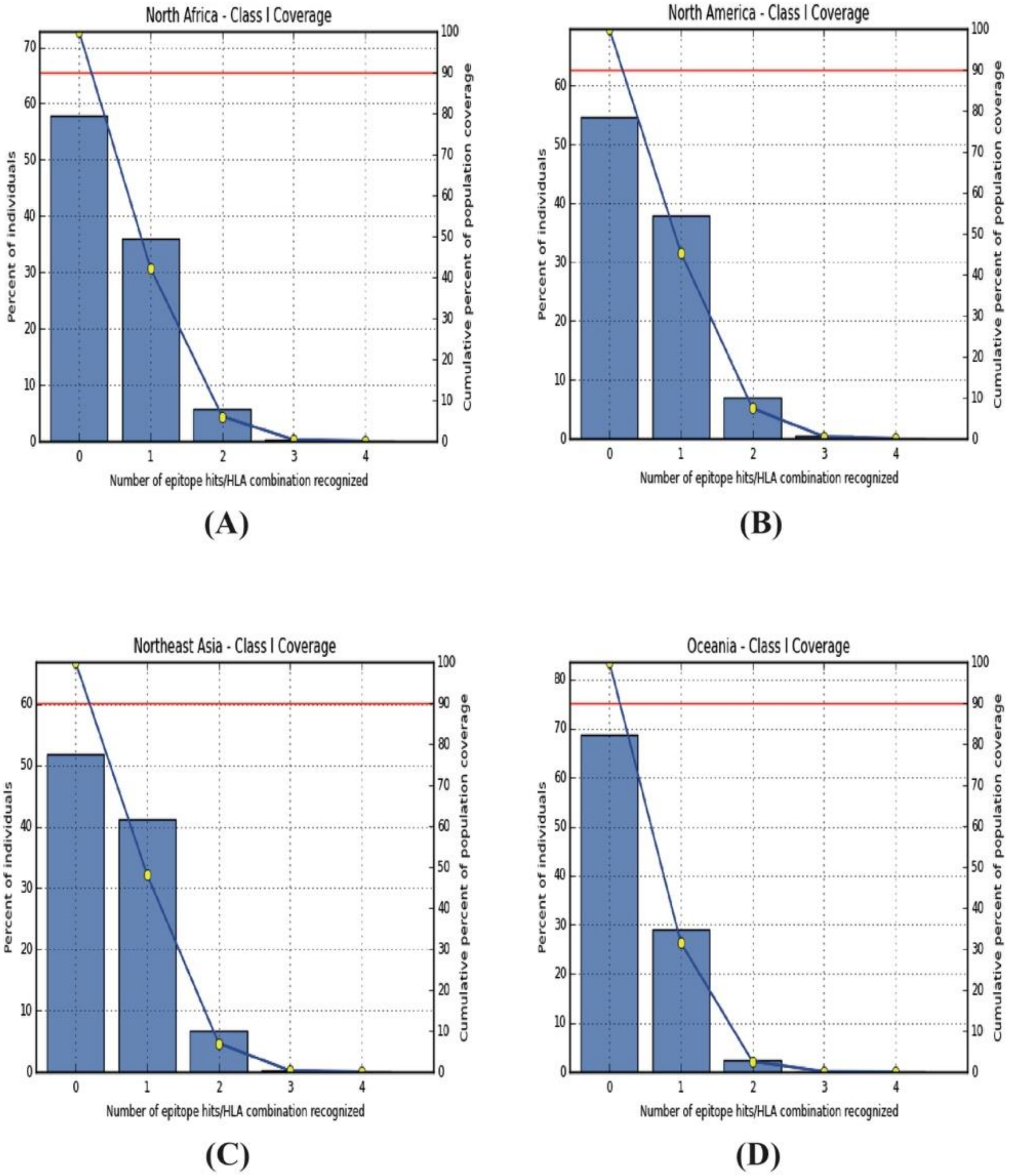

Figure S4: Population coverage based on MHC restriction data for **(A)** North Africa; **(B)** North America; **(C)** Northeast Asia; **(D)** Oceania - using the Immune Epitope Database analysis resource.

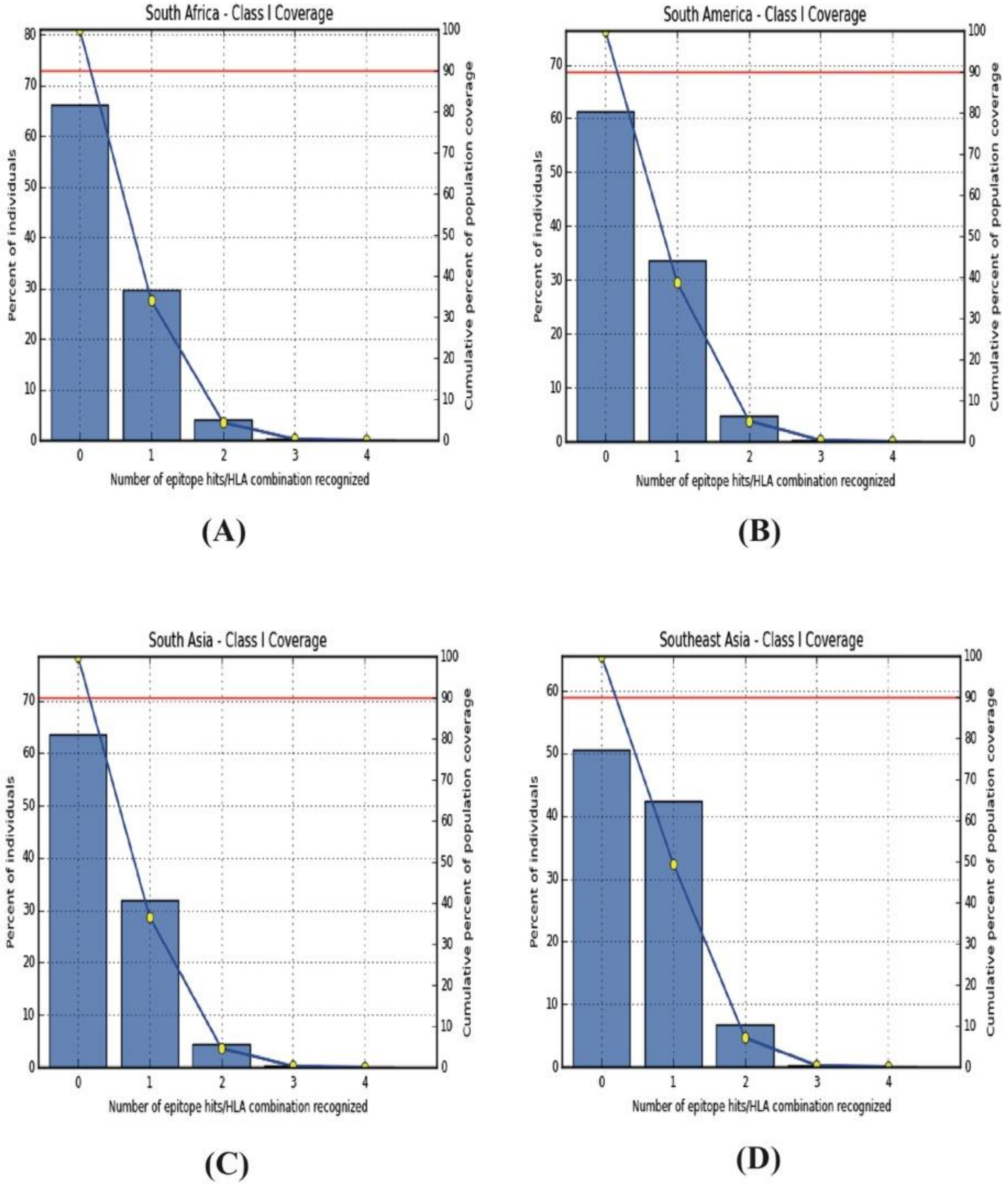

Figure S5: Population coverage based on MHC restriction data for **(A)** South Africa; **(B)** South America; **(C)** South Asia; **(D)** Southeast Asia - using the Immune Epitope Database analysis resource.

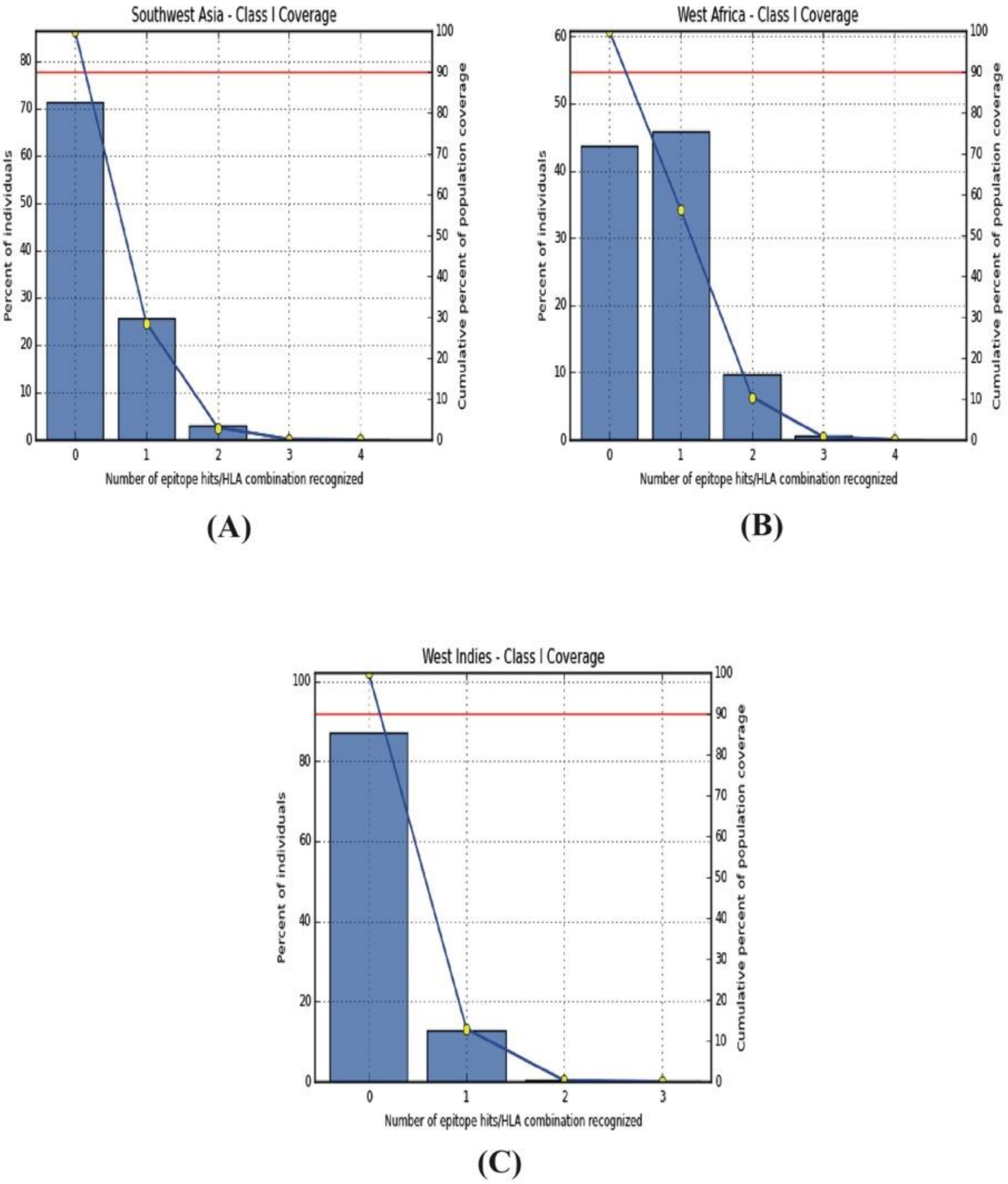

Figure S6: Population coverage based on MHC restriction data for **(A)** Southwest Asia; **(B)** West Africa; **(C)** West Indies - using the Immune Epitope Database analysis resource.
